# Avian Cholera, a Threat to the Viability of an Arctic Seabird Colony?

**DOI:** 10.1371/journal.pone.0029659

**Published:** 2012-02-15

**Authors:** Sébastien Descamps, Stéphanie Jenouvrier, H. Grant Gilchrist, Mark R. Forbes

**Affiliations:** 1 Norwegian Polar Institute, Tromsø, Norway; 2 Department of Biology, Carleton University, Ottawa, Ontario, Canada; 3 Woods Hole Oceanographic Institute, Biology Department, Woods Hole, Massachusetts, United States of America; 4 Centre d'Études Biologiques de Chizé, CNRS, Villiers-en-Bois, France; 5 National Wildlife Research Centre, Environment Canada, Ottawa, Ontario, Canada; Institut Pluridisciplinaire Hubert Curien, France

## Abstract

Evidence that infectious diseases cause wildlife population extirpation or extinction remains anecdotal and it is unclear whether the impacts of a pathogen at the individual level can scale up to population level so drastically. Here, we quantify the response of a Common eider colony to emerging epidemics of avian cholera, one of the most important infectious diseases affecting wild waterfowl. We show that avian cholera has the potential to drive colony extinction, even over a very short period. Extinction depends on disease severity (the impact of the disease on adult female survival) and disease frequency (the number of annual epidemics per decade). In case of epidemics of high severity (i.e., causing >30% mortality of breeding females), more than one outbreak per decade will be unsustainable for the colony and will likely lead to extinction within the next century; more than four outbreaks per decade will drive extinction to within 20 years. Such severity and frequency of avian cholera are already observed, and avian cholera might thus represent a significant threat to viability of breeding populations. However, this will depend on the mechanisms underlying avian cholera transmission, maintenance, and spread, which are currently only poorly known.

## Introduction

Infectious diseases are emerging at high rates [1], [2] and are thought to play a central role in species extinction or decline, loss of biodiversity and shifts in community composition [2], [3], [4], [5], [6], [7], [8]. However, a very small number of studies support this assertion [9]. Quantifications of the effects of diseases on wildlife populations are essentially at gross scale and related to changes in population numbers [10]. Even there, attributing low numbers of animal hosts to the existence of a new pathogen is problematic because this means *i)* to know the size of the host population before and after the epidemics and *ii)* to be certain that these changes are due to the disease. Moreover, disease outbreaks may affect a host population in a compensatory way [11]. For example, large die-offs due to infectious diseases have been observed in North American birds 10,12 but these die-offs usually occur on the wintering grounds. Most of the birds dying from the disease may be those already in poor condition which would have not survived migration or bred successfully in the subsequent season. This large apparent mortality on the wintering grounds may also be diluted among several distinct populations (that gather during the winter but breed at different places). Consequently, it is unclear whether or not such dramatic die-offs have a strong effect on the dynamics of local breeding populations.

A robust assessment of the effect of infectious diseases on animal populations is thus clearly needed. It needs accurate estimates of demographic parameters before and during the disease outbreak, as well as the integration of such parameters into demographic models to assess the impact of changes in reproduction and survival on population dynamics [13]. Quantifying those parameters in a free-living population is already difficult; quantifying such parameters before and during an epidemic is extremely challenging and rarely achieved. Such a lack of knowledge on the response of wild populations to diseases hampers the development of reliable predictions regarding the consequences of emergent diseases on animal populations.

Avian cholera is one of the most important infectious diseases affecting wild waterfowl, especially in North America [12]. Avian cholera is a naturally-occurring bacterial disease (*Pasteurella multocida*), reported from >150 species of wild birds, that can kill tens of thousands of birds in a single event [10], [12]. This disease currently stands out as a major problem because of the magnitude of losses it causes (usually in the wintering grounds), broad spectrum of species affected, annual frequency of epizootics, and its continually increasing geographic area of occurrence [10], [12]. Indeed, avian cholera has become widespread throughout North America [14], since the first known epizootic among North American wild ducks in 1943–44 [15].

**Figure 1:**
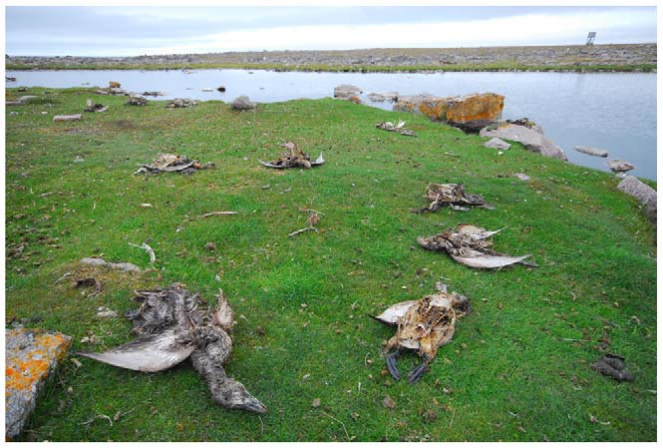
Female common eider carcasses following an avian cholera outbreak, East Bay colony, Southampton Island, Canada (photo: S. Descamps).

Since 2005, avian cholera outbreaks have occurred annually, but with different severities (i.e., the magnitude of its effect on adult female survival) on the breeding grounds of a Common eider colony located in the low Arctic, called hereafter the East Bay population [16], [17]. The observed mass mortalities were clearly the result of an infectious disease (Fig. 1). Annual laboratory analyses of a sample of eider carcasses confirmed that *P. multocida* caused the death [18], [19] and hundreds of dying eiders presented the symptoms of infection by *P. multocida* . To study the consequences of these epidemics on population dynamics of common eiders at East Bay we estimated survival and breeding parameters in relation to avian cholera, and developed a population model. We determined whether or not epidemics of avian cholera were sustainable for this colony through a stochastic modeling approach for different outbreak severities [13], [20]. We examined the effect of cholera on both the long term population growth rate and on short-term transient population projections.

## Results

**Figure 2:**
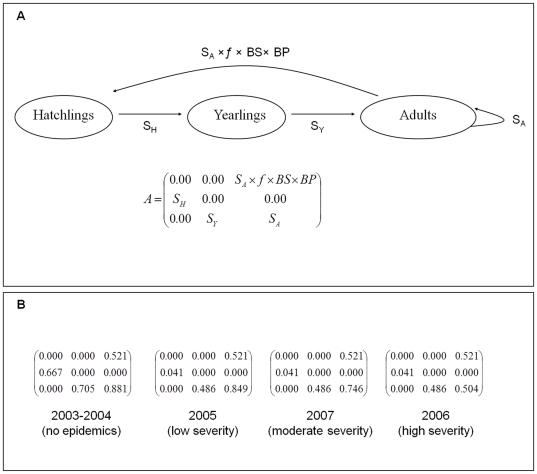
Population model for Common eiders breeding at the East Bay colony, Southampton Island, Canada. **A.** Life cycle of common eiders (Southampton Island, Nunavut, Canada) based on three age classes. The population matrix *A* contains the vital rates and projects the population from time *t* to *t*+1. The fertility parameter was calculated as the product between the breeding probability (*BP*), the average breeding success (*BS*) and the average number of hatchlings per breeding female (*f*). *S_A_* represents adult survival (survival from 2 years of age onwards), *S_Y_* survival of yearlings (from 1 to 2 years of age) and *S_H_* survival of hatchlings (from hatching to 1 year of age). We considered four different periods based on cholera severity; demographic parameters for each period are shown in **B.**

Avian cholera strongly affects survival of all age classes (Fig. 2; *Table S1*), but not breeding success (of females that did not die from cholera; Fig. 2). Adult survival was constant before the cholera years (2003–2005), but then highly variable (survival decreased by 4 to 43% in period 2005–2008; *Table S1*; Fig. 2). The survival of first year and second year individuals was constant during the periods before (2003–2005) and after (2005–2008) the first cholera epidemics (*Table S1*), but markedly dropped between these two periods (decrease of about 94% and of 31% respectively; Fig. 2). The hatching success and number of hatchlings did not vary among years (year effect on hatching success: Wald chi-square 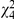 = 3.52, p = 0.48; year effect on number of hatchlings: F_4, 26_ = 1.07, p = 0.39) and averaged 0.52 (95% CI = [0.48; 0.57]) and 2.48 (95% CI = [2.05; 2.92]), respectively. Considering a two-modality variable (“before” and “during the cholera epidemics”) instead of a categorical “Year” effect did not change these results. Based on a breeding probability of 0.80 (see *Materials and Methods*, and *Text S1* for details), we thus obtained an estimated fertility of 0.521 (Fig. 2).

**Figure 3:**
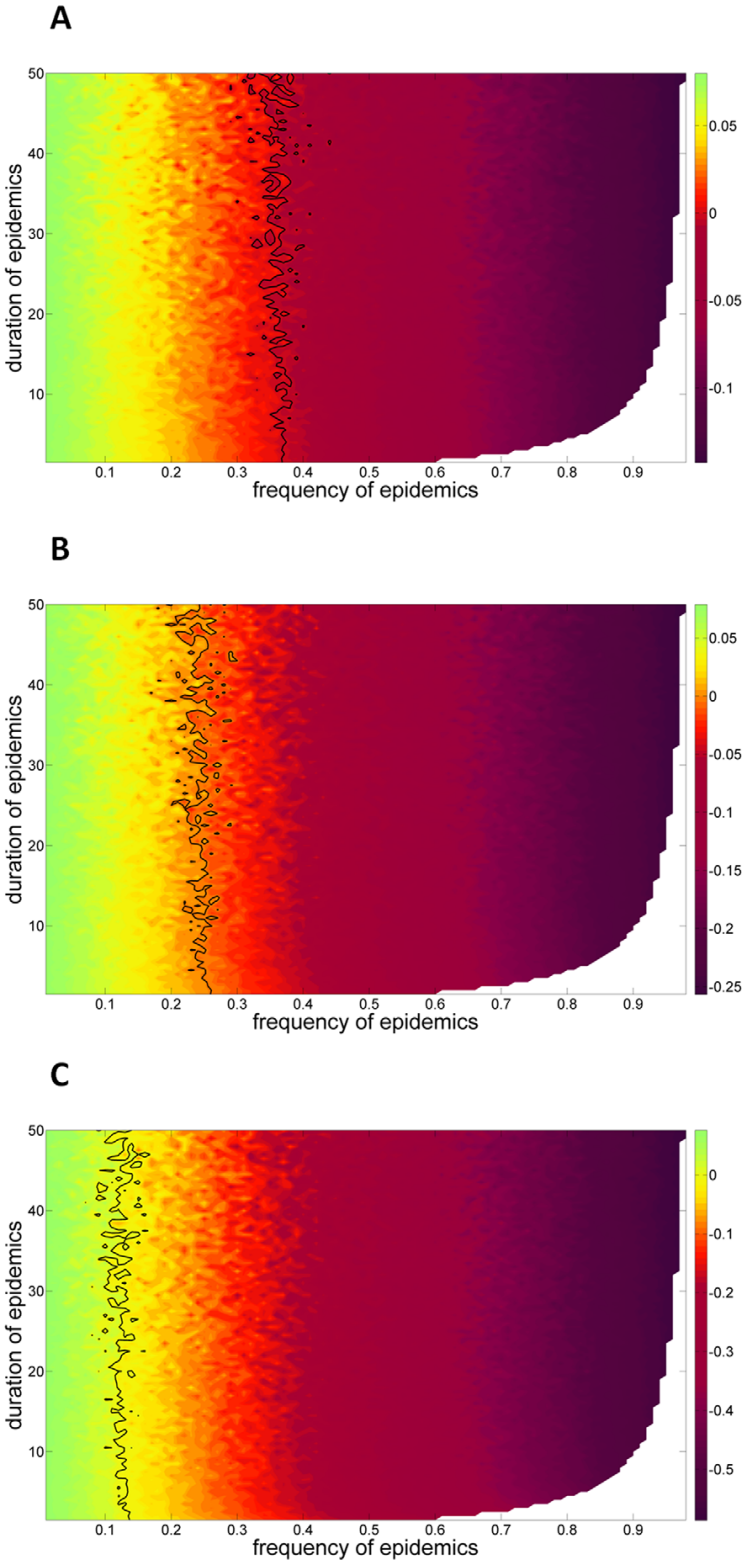
Long term stochastic growth rates of the East Bay common eider population (Southampton Island, Canada). The stochastic growth rates (*log-λ_s_*) is described as a function of the frequency (number of epidemics per decade) and duration (number of years of epidemics in a row) of avian cholera epidemics. The growth rates were calculated from a stochastic model with two states: no cholera outbreak and cholera outbreak. For the state “cholera outbreak”, we considered different severities (i.e., different level of adult mortality) of epidemics: a low severity as observed in 2005 (**A**), a moderate severity as observed in 2007 (**B**) and a high severity as observed in 2006 (**C**). The black lines denote log-*λ_s_* = 0; colors correspond to different growth rates ranging from blue (positive growth rate, log-*λ_s_*>0) to red (negative growth rate, log-*λ_s_*<0). Areas to the right of the black lines indicate combinations of epidemic frequency and duration that are not sustainable for the population (log-*λ_s_*<0). The white areas represent impossible combinations of epidemic frequency and duration.

Avian cholera strongly affects the long-term population growth rate. When considering a cholera outbreak of low severity (Fig. 2), we found that the long-term stochastic growth rate was negative for an average epidemic frequency above ∼0.35 (i.e., 3.5 outbreaks of avian cholera per decade; Fig. 3). This threshold decreased to ∼0.25 and ∼0.15 when considering moderate and severe epidemics, respectively (Fig. 3). Consequently, whatever the severity of the epidemics, more than one outbreak within three years will not be sustainable for the population. In case of severe epidemics, this threshold dropped to almost one outbreak within seven years. For a given frequency of epidemics, the duration of the epidemics, and thus the inter-annual correlation in the probability of outbreaks, had no effect on long-term population growth rate (Fig. 3).

**Figure 4:**
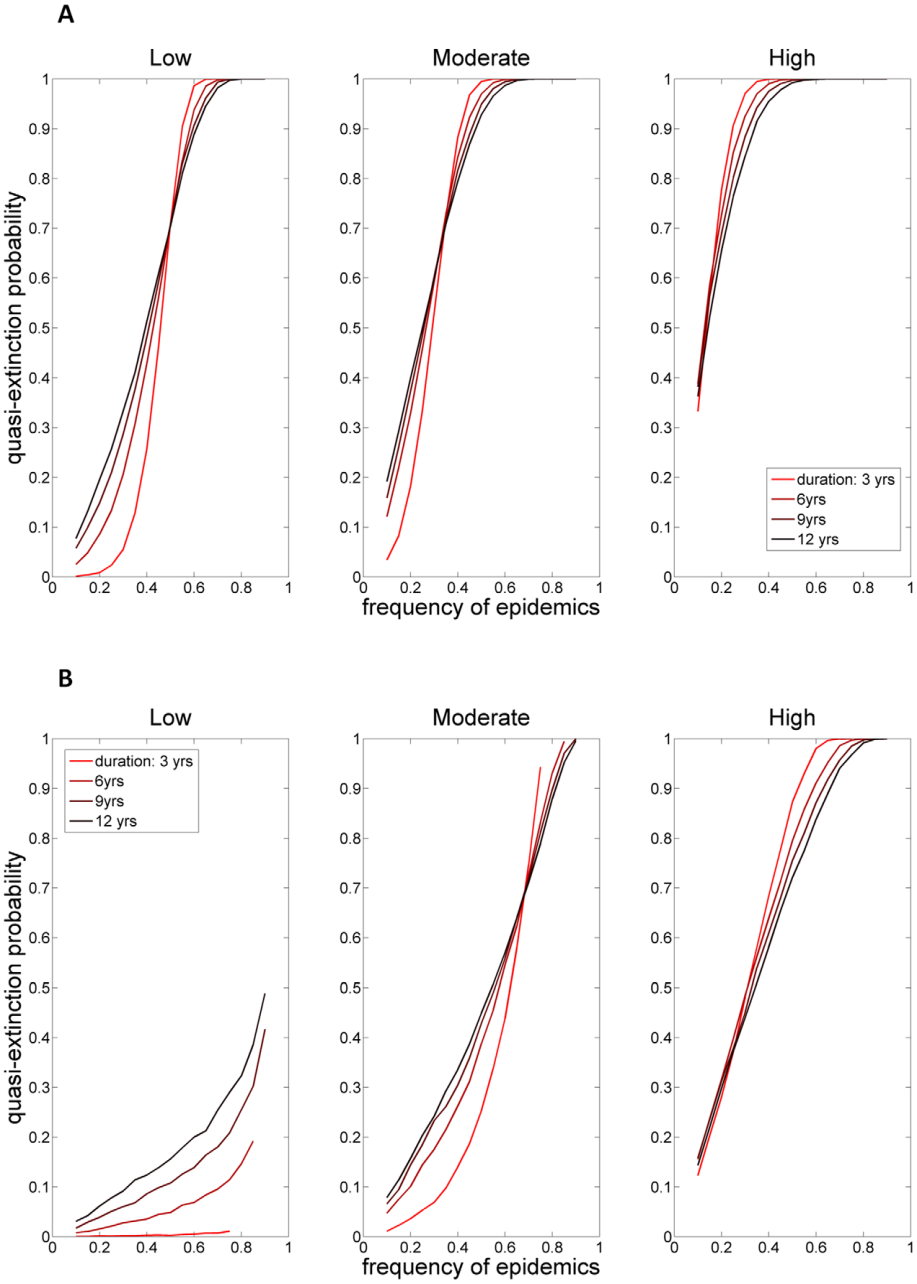
Risk of quasi-extinction for the East Bay common eider colony (Southampton Island, Canada).

The effect of avian cholera on the short-term transient population responses is similar to the one for the long-term population growth rate. In case of low, moderate or high severity epidemic, the probability that the population will decline by more than 90% within the next century (hereafter quasi-extinction probability) increased dramatically for a frequency of epidemics above 0.3, 0.2 and 0.1, respectively (Fig. 4a). A frequency of severe epidemics equal to 0.4 gives a probability of quasi-extinction close to 70% within the next 20 years (Fig. 4b). Consequently, more than four severe outbreaks per decade is very likely, even over a very short term, to lead to dramatic decline and potentially extinction of the East Bay eider population. The duration of the epidemics (number of years of epidemics in a row) did not strongly influence the risk of quasi-extinction, and this effect is essentially for low severity epidemics (Fig. 4). For epidemics of high frequency, the risk of quasi-extinction is higher for short epidemics than for long ones. The opposite is true for epidemics of low frequency. In an environment with a high frequency of short epidemics, the population experiences on average epidemics earlier than in an environment with high frequency of long epidemics. Consequently, the time for population recovering before the next epidemic is not enough, resulting in a higher probability of quasi-extinction. In an environment with low frequency of epidemics, short duration of the epidemics results in a lower probability of extinction because population trajectories are likely to increase enough before they experience an epidemic (which was not likely to happen at high frequency of epidemics).

## Discussion

Our results reveal that avian cholera should strongly affect the short-term transient dynamics and long-term growth rates of the breeding population of the Common eider at East Bay, Canadian arctic. Avian cholera in anatidae spread across United States and Canada since the seventies [10]. It has also struck several wild bird populations in marine environment of North America, Europe, Africa and even Antarctica [21]. Moreover, frequency of avian cholera seemed to be increasing [21] and now, avian cholera (with avian botulism) kills more wild waterfowl than all other diseases combined [21]. Avian cholera thus represents an emerging disease in freshwater and marine environments and could be a major problem for wildlife and biodiversity conservation.

Our study indicated that the threshold frequency of avian cholera outbreaks sustainable for the population was low in most circumstances. The duration (i.e. autocorrelation) of the epidemics has, however, no effect on the long-term stochastic population growth rate (Fig. 3). The common eiders being a long-lived species exhibits a life-history buffered against environmental autocorrelation [22]. Whatever the severity of the epidemics, a frequency above ∼0.35 (i.e., more than 1 outbreak within 3 years) will lead to population decline. In case of a severe epidemic, this threshold dropped to 0.15 (i.e., ∼1 outbreak every 7 years). The probability of quasi-extinction in the next century is >0.70 for epidemics of low severity occurring at a frequency of 0.5 (1 outbreak every other year on average), or for epidemics of high severity occurring at a frequency of 0.2 (1 outbreak every 5 years on average). The East Bay eider population could even become quasi-extinct within the next 20 years in case of a severe epidemic occurring at a frequency >0.4 (risk of pseudo extinction in this case close to 70%). Consequently, just a few outbreaks of avian cholera have the potential to lead this eider colony to extremely rapid quasi-extinction.

A frequency higher than two outbreaks of avian cholera per decade is not unusual. For example, at the île Blanche common eider colony (St-Lawrence estuary, Québec, Canada), avian cholera mortalities have been observed in seven breeding seasons during the last 27 years, which corresponds to a frequency of 0.26, and the size of the colony is declining (J.-F. Giroux, *pers. comm*.). In South-Africa, avian cholera impacted a cormorant colony at a frequency of 0.3 between 1991 and 2005 [23], but no clear trend is detected yet in this colony. For these two examples, the impact of cholera on vital rates is unknown so that it is unclear whether or not such frequency of avian cholera epidemics should have led to population decline. In United States, several waterfowl wintering areas suffered avian cholera epidemics at a frequency >0.3 during the last 15 years (source: http://www.nwhc.usgs.gov/publications/quarterly_reports/index.jsp), and there are even some areas where the occurrence of avian cholera was annual [12]. All these examples show that recurrent and frequent epidemics of avian cholera are common.

Our predictions do not take into account potential adaptations of individuals that could occur through microevolution or phenotypic plasticity and/or through an increase in the % of resistant individuals. Considering the long-generation time for common eider, it seems very unlikely that they will adapt quickly enough to cope with this emergent disease if its frequency/severity remains as high as what has been observed since 2005. An adaptation through phenotypic plasticity would mean that eiders adjust their behavior in response to the disease, e.g, by skipping the reproductive season when an epidemic occurs or by decreasing clutch size which may increase survival [16]. But what proximal mechanisms would trigger such changes in reproductive behavior? There is no clear and straightforward answer and an adaptive response through phenotypic plasticity does not seem very likely. Finally, an increase in the proportion of resistant individuals within the population also seems unlikely, or at least, we have no evidence that the proportion of resistant individuals increased following recurrent outbreaks at the East Bay colony. Indeed, the severity of the outbreaks did not show any sign of decrease since the first outbreak in 2005 and was similar, and very high, in 2008 and 2006, when >30% of breeding females died from the disease [18], [19].

Moreover, our study did not take into account possible density-dependence in eider survival or reproduction. After an epidemic of cholera, severe reductions in population size might mean recovery is faster than what our study predicted if survival and reproduction also increase beyond estimates included in the model. However, density dependence was likely weak in 2003–2004, which is the period used to define “normal” or non-cholera years. Indeed, the East Bay population was still increasing when cholera struck this population. Duckling survival was also very high at this time [24]. Consequently, even if we cannot reject the potential role of density dependence in eider vital rates, our predictions about the effect of avian cholera on eider extinction risk seem rather conservative and nothing suggests that after an epidemic, recovery would be faster that what our model predicted. How likely diseases are to drive their host populations to extinction likely depend on many factors including population naivety, density-dependent transmission or whether other species vector the disease. Indeed, once the disease has reduced its host population to a certain level, it should be unable to transmit to new individuals in single host species contexts, because of a too low density. The disease should thus become extinct before its host. This view is however predicated on the idea that disease transmission is density-dependent, which is not necessarily the case [25]. For example, in our system, transmission of avian cholera between eiders may occur when they use the main pond located in the colony [26]. Only a few healthy carriers, that are not necessarily eiders but maybe herring gulls or snow geese [27], could contaminate this pond (by drinking and/or cleaning themselves). Then, as all breeding eiders do use this pond at some points during the breeding season, transmission of the disease among eiders is possible and may lead to an epidemic outbreak even at very low eider density. This agrees with field observations indicating that the severity of the epidemics (its impact on breeding female mortality) was not related to the density of breeding females [18]. This potentially density-independent disease transmission, combined to the fact that avian cholera is likely an evolutionary novel pathogen (and thus that eiders have not evolved any behavioral or physiological adaptations in face of such epidemics), may create optimal conditions for a disease to drive population extinction. However we should note that, even if we did not detect any relationship between eider density and the magnitude of avian cholera outbreaks, there might be some specific host density threshold below which avian cholera may not invade the population.

We recognize that our conclusions are based on a simple population model and remain theoretical since the eider population has not been extinct yet. However, the dramatic decline in the number of breeding females observed at the East Bay colony supports our conclusion that avian cholera can, at least, cause a very strong depletion in the host population. Such an impact is mainly the consequence of a strong effect of cholera on adult survival, which is the parameter of highest sensitivity in long lived birds [28], including eiders, Before concluding with certainty that avian cholera will lead to the extinction of the East Bay eider population, one would need to add an epidemiological dynamics component into our model. Indeed, one important assumption of our models is that the demographic effect(s) of cholera epidemics will be all outbreaks. To test whether this assumption is valid or not, we need to understand the underlying mechanisms of the emergence, persistence and severity of the disease. This will help to answer the following questions: why does an outbreak occur? Why is this outbreak more or less severe? Can individuals get acquired immunity, and if yes, in which proportion? Even if it is clear from our study that avian cholera can cause a steep population decline, all these questions need to be answered to determine whether or not it will lead to population extinction. Virtually nothing is known about dynamics of avian cholera outbreaks and its transmission. Further research is clearly needed into those directions to understand what determines disease frequency, severity and spread.

**Figure 5:**
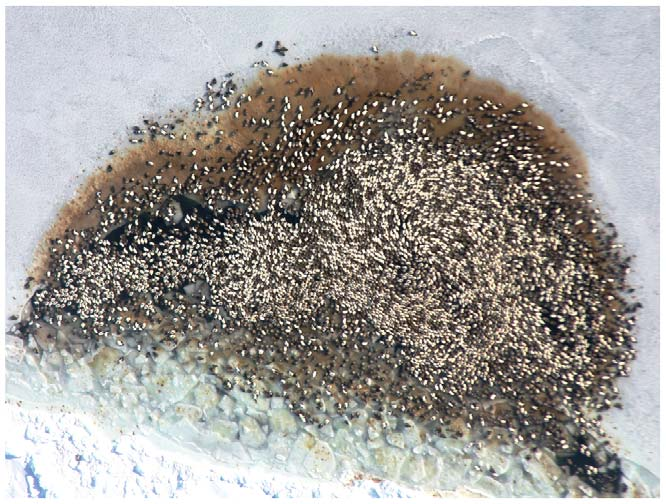
Winter aggregation of common eiders in North Atlantic (photo: H.G. Gilchrist).

Diseases in general and avian cholera in particular, remain a relatively under-studied topic in conservation biology relative to their expected influence. In time of climate warming, infectious diseases might represent an important threat to biodiversity as their occurrence and impacts may be linked to temperature [1], [2], [29], [30], [31]. Our study emphasized the importance of disease severity and outbreak frequency. Inter-connectivity between colonies or populations is also an important factor to consider. Indeed, dispersal of potentially infectious individuals, i.e. healthy carriers [27], might be of paramount importance in the spread of the disease. Indeed, individual dispersal and/or dense aggregation in winter time (Fig. 5) might clearly favor the spread of the disease to other colonies, and eventually other countries. This should be taken into account when modeling and predicting the risk that avian cholera represents at a wide geographical scale. In this context, a better understanding of diseases dynamics and impact on animal populations is needed, as is a careful monitoring of the presence of diseases, especially at high latitudes [32], where environment are clearly at risk.

## Materials and Methods

### Study population

Our study was conducted in the largest eider colony in Canadian arctic from 2003 to 2009 [16], [17]. This colony is located on a small rocky island (Mitivik Island; 0.24 km^2^) within the East Bay Migratory Bird Sanctuary, Southampton Island, Nunavut (64°02′N, 81°47′W) and comprises between 4000 and 8000 breeding pairs depending on the year. It is located >150 km away from other eider colonies and female fidelity to their breeding site is very high. The avian cholera epidemics that occurred in this colony were classified as low in 2005, severe in 2006 and moderate in 2007 based on the number of carcasses found at the end of each breeding season adjusted for the estimated total number of breeding pairs [18], [19].

### Stochastic model

The life-cycle of eiders was based on three age-classes: hatchlings, yearlings (1 year old individuals) and adults (≥2 years old). The population matrix *A* projects the population vector *n* that gives the number of individuals in each age class from time *t* to *t+1*: *n_t+1_* = *A n_t_*_._ eq 1. We parameterized female-only transition matrices *A* (Fig. 2) according to a birth-pulse post-breeding census design [13].

Survival parameters were estimated through modeling of capture-mark-recapture data of 536 adults and 492 hatchlings, and reproductive parameters (hatching success and brood size at hatching) through monitoring of 466 females of known hatching success and 31 females of known brood size (see *Text S1* for details). Note that the small sample size for estimating brood size at hatching (and thus the uncertainty around this estimate) is not problematic in our study as variation in brood size at hatching does not strongly affect eider population growth rates, due to a very low sensitivity.

Breeding probability of female common eiders is unknown and we considered the average value of breeding probabilities of 80% observed in a European population of common eiders [33]. We performed our analyses with other values of breeding probability (0.6 and 1; *Text S1*), but whatever the breeding probability considered, results were very similar.

To study the effect of avian cholera epidemics on eider population dynamics, we constructed a stochastic model where environment was assumed to be in one of the two following states: “normal year” or “year with cholera”. We considered the projection matrices previously defined as representing the “normal years” (*A_no cholera_*) and “cholera years” (*A_cholera_*), respectively. For the matrix representing the cholera years, we considered three scenarios where the matrix corresponded to epidemics of low, moderate or high severity (Fig. 2b). Then, at each time step of the growth rate calculation process, a matrix (*A_no cholera_* or *A_cholera_*) is selected according to a Markov chain with the transition matrix:
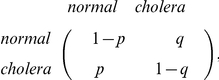
where *q* represents the transition probability from a normal to a cholera year and *p* from a cholera to a normal year. The long-term frequency and the average duration (i.e., average number of years of epidemics in a row, which reflects the auto-correlation of the epidemics episodes) of cholera outbreaks are respectively: 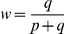 and 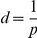 [13].

To examine the long-term effects of avian cholera epidemics on eider population dynamics, we determined the stochastic growth rate with the formula:
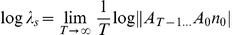
where *A_i_* represent *A_no cholera_* or *A_cholera_* [13].

To evaluate short-term transient population responses we projected transient population trajectories using eq 1 and an initial population made of 4000 adult females which corresponds approximately to the size of the breeding population in 2008. The initial number of individuals in juvenile age classes was calculated assuming a stable age distribution (determined as the right eigenvector of matrix *A_no cholera_*).

A population trajectory is defined as quasi-extinct if the population declines by more than 90%. We calculated the probability of quasi-extinction over next 20 or 100 years as the proportion of population trajectories that fall below 400 breeding pairs over the period considered. For each frequency, duration, and severity of the epidemics, we simulated 10,000 population trajectories.

## Supporting Information

Table S1 **Survival modelling of adult and juvenile female common eiders breeding at the East Bay colony, Southampton Island, Nunavut, Canada.**
(DOC)

Text S1 **Estimation of vital rates for common eiders breeding at the East Bay colony, Southampton Island, Nunavut, Canada.**
(DOC)
