# Supplementary material for: Avian Cholera, a Threat to the Viability of an Arctic Seabird Colony?
Source: PLoS One. 2012 Feb 15;7(2):e29659. doi: 10.1371/journal.pone.0029659 (PMC3280243; doi:10.1371/journal.pone.0029659)
Supplement: Table S1 — Survival modelling of adult and juvenile female common eiders breeding at the East Bay colony, Southampton Island, Nunavut, Canada. (DOC) [file pone.0029659.s001.doc]

**Table S1. *Survival modelling of adult and juvenile female common eiders breeding at the East Bay colony, Southampton Island, Nunavut, Canada***

Adult survival modelling (a) was based on both live encounters and band recoveries data (*r* represents the recovery rate and *F* the probability of fidelity to the sampling region, fixed to 1 ; see *Text-S1* for details) ; juvenile survival modelling (b) was based on live encounters only. In both cases, *ϕ* represents the survival probability and *p* the probability of recapture. *np* indicates the number of estimated parameters for each model, *AICc* is the Akaike information criterion corrected for small sample sizes and *∆AICc* the difference in AICc between the model with lowest AICc and the model considered. Subcript *t* represents the time effect; subscripts “03-05, 05-08” and “04-05, 06-08” represent two-modality variables constraining the survival and recapture parameters, respectively, to be constant in period before and during the cholera epidemics. Subscript *a* represents an age effect with 3 classes (hatching-1 year old, 1-2 years old, and >2 years old) and subscripts “*a_1_*”, “*a_2_*” and “*a_3_*”represent these 3 classes.

| **Model** | **np** | **Deviance** | **AICc** | **ΔAICc** |
| --- | --- | --- | --- | --- |
| $\varphi_{03-05, t}$ $F$ $r_{t}$ $p_{04-05, 06-08}$ | 12 | 2808.476 | 2832.791 | 0.000 |
| $F$ $r_{t}$ $p_{04-05, 06-08}$ | 13 | 2808.443 | 2834.810 | 2.019 |
| $F$ $r_{t}$ $p_{t}$ | 18 | 2800.707 | 2837.400 | 4.609 |
| $F$ $r_{t}$ $p$ | 12 | 2816.529 | 2840.843 | 8.052 |
| $F$ $r$ $p_{04-05, 06-08}$ | 11 | 2835.255 | 2857.520 | 24.729 |
| $\varphi_{03-05, 05-08}$ $F$ $r_{t}$ $p_{04-05, 06-08}$ | 9 | 2855.731 | 2873.911 | 41.120 |
| $\varphi$ $F$ $r_{t}$ $p_{04-05, 06-08}$ | 8 | 2892.793 | 2908.937 | 76.146 |

(b)

| **Model** | **np** | **Deviance** | **AICc** | **ΔAICc** |
| --- | --- | --- | --- | --- |
| $\varphi_{03-05, 05-08}^{a_{1}} \varphi_{03-05, 05-08}^{a_{2}} \varphi_{t}^{a_{3}} p$ | 11 | 744.438 | 766.873 | 0.000 |
| $\varphi_{03-05, 05-08}^{a_{1}} \varphi_{t}^{a_{2}} \varphi_{t}^{a_{3}} p$ | 14 | 738.192 | 766.887 | 0.014 |
| $\varphi_{t}^{a} p^{a}$ | 17 | 734.028 | 769.047 | 2.174 |
| $\varphi_{t}^{a_{1}} \varphi_{03-05, 05-08}^{a_{2}} \varphi_{t}^{a_{3}} p$ | 14 | 740.911 | 769.606 | 2.733 |
| $\varphi_{t}^{a} p_{t}^{a}$ | 23 | 729.987 | 777.843 | 10.970 |
| $\varphi_{t}^{a} p_{t}$ | 20 | 755.133 | 796.538 | 29.665 |
| $\varphi_{t} p^{a}$ | 9 | 785.612 | 803.908 | 37.035 |
| $\varphi_{t}^{a} p$ | 15 | 800.054 | 830.850 | 63.977 |
| $\varphi^{a} p^{a}$ | 6 | 845.184 | 857.322 | 90.449 |
| $\varphi p^{a}$ | 4 | 847.882 | 855.947 | 89.074 |
