# Supplementary material for: Avian Cholera, a Threat to the Viability of an Arctic Seabird Colony?
Source: PLoS One. 2012 Feb 15;7(2):e29659. doi: 10.1371/journal.pone.0029659 (PMC3280243; doi:10.1371/journal.pone.0029659)
Supplement: Text S1 — Estimation of vital rates for common eiders breeding at the East Bay colony, Southampton Island, Nunavut, Canada. (DOC) [file pone.0029659.s002.doc]

**Text S1. *Estimation of vital rates for common eiders breeding at the East Bay colony, Southampton Island, Nunavut, Canada***

*Juvenile and adult survival*

Our study was conducted at the East Bay eider colony from 2003 to 2009. Birds (adult females and juveniles at hatching) were banded from 2003 to 2008, and resighting occured from 2003 onward. Adult females were banded with metal and color rings, as well as two plastic temporary nasal tags (plastic markers attached with absorbable suture monofilament). Ducklings were banded with both an aluminum ring and a small color plastic ring (details in [1])

Analyses of eider survival were carried out using Capture-Mark-Recapture methods [2], implemented in the software MARK [3]. We analyzed separately survival of adult females (individuals of unknown age) and survival of juveniles (juveniles banded at hatching). Indeed, by considering only known-age individuals, we would not be able to get estimates of adult individuals before the cholera epidemic (i.e. before 2005). Moreover, adult survival modelling was based on both re-sighting and band recoveries data (238 bands among our sample of 536 females were recovered, on the breeding grounds, on carcasses during cholera epidemics), whereas juvenile survival modelling was based on re-sighting (live encounters) data only. Annual adult survival could be estimated for period 2003-2008 [2]. As most juveniles come back to the colony for the first time at two years of age [1,4,5], survival from hatching to one year of age could be estimated during 2005-2008.

Duckling sex was unknown at banding. As males do virtually not come back to their natal breeding site whereas females do [4,6,7], estimating survival rates using all ducklings banded at hatching will underestimate true survival. Consequently, to get a more relevant estimate of duckling survival from hatching to one year of age, we removed half of the individuals from our sample of banded ducklings assuming that sex-ratio at hatching was 1:1 [8]. We removed only individuals not re-sighted again, assuming they were males. Our juvenile survival analyses were then based on n=492 ducklings (among which, 92 were re-sighted at the colony).

Adult survival modelling was based on both live encounters and band recoveries data. In such a framework, the fate of an individual is governed by probabilities *S* (true survival probability), *r* (recovery rate), *F* (probability of fidelity to the sampling region), and *p* (probability of recapture, conditional on being alive and in the sampling region). Here, recoveries correspond to bands recovered on carcasses during cholera epidemics and all dead recoveries and live encounters occur in the same sampling area. Consequently, the fidelity *F* was fixed to 1. As no recovery occurred before the cholera epidemics, *r* was fixed to 0 for years 2003 and 2004.

Model selection was based on a variant of the Akaike Information Criterion corrected for small sample size [AICc, 9], using ∆AICc (difference in AICc between a given model and the model with lowest AIC). A ∆AICc<2 between two competing models means that they cannot be distinguished in their ability to model the data [9]. When two nested models were characterized by ∆AICc<2, the simplest one was preferred. As survival rates were the parameters of interest in our study, we first modelled re-sighting (and recovery rates for adult females) to have more statistical power when modelling survival [2]. We started our model selection process from the general models $\varphi_{t} F r_{t} p_{t}$ for adult female survival modelling and $\varphi_{t}^{a} p_{t}^{a}$for ducklings survival modelling (*t* and *a* indicate *time* and *age* variations, respectively). Age was defined as a three modality variable (hatching to one year of age, one to two years of age and two years of age onwards).

For adult data (mixture of dead recoveries and live encounters), the goodness-of-fit test of the general model $\varphi_{t} F r_{t} p_{t}$ was performed with the bootstrap procedure implemented in software MARK [3], using 1000 simulations. This test indicated an acceptable fit (p=0.274), so that the “*iii*” assumption (i.e., independence of fates and identity of rates among individuals), required for CMR analyses, was met. For juvenile data (i.e., live encounters only), survival and recapture probabilities are clearly age-dependent [5, this study], rejecting thus the “iii” assumption. To verify that once recruited, data on known-age individuals support this assumption, we conducted the GOF test over the capture histories of the birds with first captures removed. This test indicated a good fit with the CJS model ($\chi_{9}^{2}$=2.81, p=0.97). The age structure associated to recruitment (3 age classes) was included in our capture-recapture models (see Table S1).

*Reproduction*

Breeding status of birds banded as juvenile and re-sighted at the colony was unknown so that we were unable to assess age at which first breeding occurs in our population. We thus considered that first breeding occurs at two years of age, as generally observed in common eiders [4,5]. This is in agreement with our observations that most of the birds banded as ducklings were re-sighted for the first time at two years of age (63% of first re-sightings of individuals banded at hatching). Breeding probability was also unknown because the breeding status of a large proportion of banded birds was unknown. We thus considered the average value of breeding probabilities of 80% observed in a European population of common eiders [10]. To our knowledge, this represents the only available estimate of breeding probability in common eiders. Based on the uncertainty of this parameter, we performed our analyses with different values of breeding probability (0.6 and 1).

Annual breeding success of eiders was estimated through direct observations of breeding nasal-tagged females. A nest was considered as successful when females have been seen on the nest for at least 24 days continuously [the average incubation length, 11] and/or when female have been seen with ducklings in the nest. We only considered females of known breeding success and who did not die from cholera during the incubation period (n=466). And finally, the number of hatchlings per nest was estimated through direct observations of 31 females leaving their nests just after hatching. We determined whether or not these breeding parameters showed significant inter-year variations with a logistic regression and an ANOVA to test for a year effect on average breeding success and average number of hatchlings, respectively (software SAS v.9).

**References**

1. Descamps S, Forbes MR, Gilchrist HG, Love OP, Bêty J (2011) Avian cholera, post-hatching survival and selection on hatch characteristics in a long-lived bird. Journal of Avian Biology 42: 39-48.

2. Lebreton J-D, Burnham KP, Clobert J, Anderson DR (1992) Modeling survival and testing biological hypotheses using marked animals: a unified approach with case studies. Ecological Monographs 62: 67-118.

3. White GC, Burnham KP (1999) Program MARK: Survival estimations from populations of marked animals. Bird Study 46 (Suppl.): 120-138.

4. Goudie RI, Robertson GJ, Reed A (2000) Common Eider (*Somateria mollissima*). In: Poole A, Gill F, editors. The Birds of North America, No 546. Philadelphia, Pennsylvania: The Academy of Natural Sciences.

5. Baillie SR, Milne H (1982) The influence of age on breeding in the Eider *Somateria mollissima.* Bird Study 29: 55-66.

6. Bustnes JO, Erikstad KE (1993) Site fidelity in breeding common eider *Somateria mollissima* females. Ornis Fennica 70: 11-16.

7. Swennen C (1990) Dispersal and migratory movements of eiders *Somateria mollissima* breeding in the Netherlands. Ornis Scandinavica 21: 17-27.

8. Swennen C, Duiven P, Reyrink LAF (1979) Notes on the sex-ratio in the Common Eider *Somateria mollissima* (L.). Ardea 67: 54-61.

9. Burnham KP, Anderson DR (2002) Model selection and multimodel inference: a practical information-theoretic approach. New York: Springer-Verlag.

10. Coulson JC (1984) The population dynamics of the Eider Duck *Somateria mollissima* and evidence of extensive non-breeding by adult ducks. Ibis 126: 525-543.

11. Bottita GE, Nol E, Gilchrist HG (2003) Effects of experimental manipulation of incubation length on behavior and body mass of common eiders in the Canadian Arctic. Waterbirds 26: 100-107.
